# 

**Published:** 1878-07

**Authors:** 


### BOOKS AND PAMPHLETS RECEIVED.

Fluid Extracts by Percolation. By Edward R. Squibb, M. D., Brooklyn, N. Y. Reprint from *Amer. Jour. of Phar.* for May, 1878, with additions by the author.

Metric Weights and Measures for Medical and Pharmacal Purposes. Published by the Government for Official Service.

The Intravenous Injection of Milk as a Substitute for Transfusion of Blood· Illustrated by seven operations. By T. Gaillard Thomas, M. D., New York. Reprint from the *New York Med. Jour.*, May, 1878.

A Contribution to the Therapeutics of Medicine. Read before the Section on Practice of Medicine in the New York Academy of Medicine, Nov. 20, 1877. By E. C. Seguin, M. D., President of the New York Neurological Society.

Lapar's Elytrotomy; A Substitute for the Cæsarean Section. By T. Gaillard Thomas, M. D., New York. Reprint from the *American Journal of Obstetrics and Diseases of Women and Children*, Vol. XI., No. 11, April 1878.

Second Annual Report of the State Board of Health of Wisconsin, for the year ending December 31, 1877.

Sterility and its Treatment. By William H. Wathen, M. D., Clinical Lecturer on Diseases of Women and Children, Louisville Medical College, etc., etc. Reprint from the Transactions of the Kentucky State Medical Society, 1877.

Amputation of Cervix Uteri. By W. H. Wathen, M. D., Clinical Lecturer on Diseases of Women and Children, Louisville Medical College, etc., etc. Read at the Kentucky State Medical Society, April 3, 1878. Reprint from May No. of *Richmond and Louisville Medical Journal.*

Comparison of the Results ot the Cæsarean Section and Lapar's Elytrotomy in New York. By T. Gaillard Thomas, M. D. Reprint from the *New York Medical Journal*, May, 1878.

A New Treatment for Spine Diseases. By Meigs Case, M. D., Oneonta, N. Y. Reprint from the *Cincinnati Lancet and Observer*, May, 1878.

Suicide Not Evidence of Insanity. Read before the Medico-Legal Society of the City of New York, March, 1878. By Hon. H. O. Palmer, of New York.

Original Lectures—Tumor of the Male Breast and Cyst of the Neck. By J. H. Pooley, M. D., Professor of Surgery, Sterling Medical College. Reprint from the *Ohio Medical and Surgical Journal.*

Physics of the Infectious Diseases, Comprehending a Discussion of certain Physical Phenomena in connection with the Acute Infectious Diseases. By C. A. Logan, A. M., M. D. Published by Jansen, McClurg & Co., Chicago, 1878.

### ANNOUNCEMENTS FOR THE MONTH.

### SOCIETY MEETINGS.

Chicago Medical Society—Mondays, July 8 and 22.

Chicago Society of Physicians and Surgeons—Mondays, July 1 and 15.

### CLINICS.

MONDAY.

Eye and Ear Infirmary—2 to 4 p. m., by Prof. Holmes and Dr. Hotz—2 p. m., Prof. Jones.

Mercy Hospital—2 to 3 p. m. Surgical, by Prof. Andrews.

Rush Medical College—2:30 p. m. Dermatological and Venereal, by Dr. Hyde.

County Hospital—8 p. m. Necropsy, by Dr. Danforth.

Woman's Medical College—3 p. m. Surgical, by Prof. Owens.

TUESDAY.

County Hospital—1:30 p. m. Medical, by Prof. Lyman ; 2:30 p. m. Surgical, by Prof. Parkes.

Mercy Hospital—2 p. m. Medical, by Prof. Hollister.

Eye and Ear Infirmary—2 p. m. Prof. Jones.

WEDNESDAY.

County Hospital—2 p. m. Gynecological, by Dr. Bridge. 3 p. m. Ophthalmological, by Dr. Montgomery.

Mercy Hospital—2 p. m. Eye and Ear, by Prof. Jones.

Rush Medical College—4 p. m. Diseases of the Chest, by Dr. E. Fletcher Ingals.

THURSDAY.

Mercy Hospital—2 p. m. Medical, by Prof. Davis.

Rush Medical College—1:30 p. m. Neurological, by Prof. Lyman.

Eye and Ear Infirmary—2 to 4 p. m. Operations by Prof. Holmes and Dr. Hotz.

FRIDAY.

Mercy Hospital—2 p. m. Medical, by Prof. Davis.

County Hospital—1:30 p. m. Medical, by Prof. Quine ; 2:30 p. m., Surgical, by Prof. Powell.

Woman's Medical College—10 p. m. Ophthalmological, by Dr. Montgomery.

SATURDAY.

Rush Medical College—2 p. m. Surgical, Prof. Gunn.

Chicago Medical College—2 p. m. Surgical, by Prof. Andrews and Isham ; 3 p. m., Diseases of the Chest, by Prof. Johnson.

Woman's Medical College—12 m. Gynecological, by Prof. Fitch ; 3 p. m. Dermatological, Dr. Maynard.

Special Clinics daily, from 2 to 4 p. m., at the South Side Dispensary, and at the Central Free Dispensary.

